# *Oreocharis oriolus*, a new species of Gesneriaceae in a sclerophyllous oak community from Yunnan, Southwest China

**DOI:** 10.1002/ece3.10174

**Published:** 2023-06-26

**Authors:** Jun Hu, Jun‐Yi Zhang, Hai He, Ding‐Xiang Yu, Hong Jiang, Qing Liu, Fang Wen

**Affiliations:** ^1^ CAS Key Laboratory of Mountain Ecological Restoration and Bioresource Utilization & Ecological Restoration and Biodiversity Conservation Key Laboratory of Sichuan Province, Chengdu Institute of Biology Chinese Academy of Sciences Chengdu China; ^2^ University of Chinese Academy of Sciences Beijing China; ^3^ College of Life Sciences Chongqing Normal University Chongqing China; ^4^ Institute of Feature Crops Chongqing Academy of Agricultural Sciences Chongqing China; ^5^ Guangxi Key Laboratory of Plant Conservation and Restoration Ecology in Karst Terrain Guangxi Institute of Botany, CAS Guilin China; ^6^ National Gesneriaceae Germplasm Resources Bank of GXIB, Gesneriad Committee of China Wild Plant Conservation Association, Gesneriad Conservation Centre of China (GCCC) Guilin Botanical Garden, CAS Guilin China

**Keywords:** endemic species, flora of China, Gesneriaceae, *Oreocharis forrestii*, *Oreocharis georgei*, phylogeny, taxonomy

## Abstract

*Oreocharis oriolus*, a new species of Gesneriaceae in a sclerophyllous oak community from Yunnan, Southwest China, is described and illustrated. Morphologically, it resembles both *O. forrestii* and *O. georgei*, while it is distinct in combined characters of wrinkled leaves, peduncle and pedicel covered with whitish and egladular villous hairs, bract lanceolate and nearly glabrescent adaxially, and staminode absent. Molecular phylogenetic analysis based on nuclear ribosomal internal transcribed spacer (*nr*ITS) and chloroplast DNA fragment (*trnL‐F*) of 61 congeneric species also supported *O. oriolus* as a new species while it was nested with *O. delavayi*. It was currently assessed as ‘Critically Endangered’ (CR) regarding to its small‐sized population and narrow distribution following the IUCN categories and criteria.

## Graphical abstract

*Oreocharis oriolus*, a new species of Gesneriaceae in a sclerophylla oak community from Yunnan, Southwest China, is described and illustrated. It was currently assessed as ‘Critically Endangered’ (CR) regarding to its small‐sized population and narrow distribution following the IUCN categories and criteria.

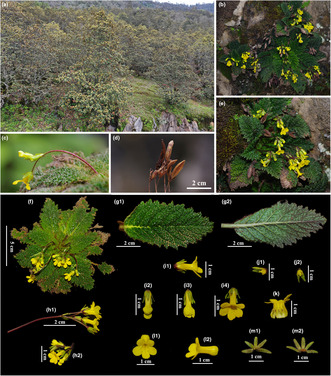

## 1 INTRODUCTION

*Oreocharis* Benth. in Bentham & Hooker (1876: 995) is a genus in Gesneriaceae consisting of ca. 29 species with 28 species and five varieties documented in Chinese floras (Li & Wang, 2005; Wang et al., 1998). However, with the reclassification of the genera in light of the extensive molecular phylogenetic and morphological studies of this family (Möller et al., 2011a, 2011b; Möller et al., 2014, Möller et al., 2015; Middleton et al., 2013; Chen et al., 2014; Yang et al., 2020), the enlarged concept of *Oreocharis* included ca. 140 accepted names (GRC, 2022). By and large, the majority taxa of this genus were found in the South (Fujian, Guangdong, Guangxi, Hainan, Hubei, Hunan and Jiangxi) and Southwest (Guizhou, Sichuan, Yunnan, Xizang and southern Gansu) China, while few species also extending to North Vietnam, Myanmar, Bhutan, India, Japan, and Thailand (Chen et al., 2018; Do et al., 2017; Möller et al., 2017, 2018; Wei, 2018; Wei et al., 2022; Xuyen et al., 2016).

Quite a few species in *Oreocharis* were found on cliffs or in crevices of mountainous areas, where it is usually difficult to access (Wang et al., 1990, 1998). Recently, with explorations accessible to formerly remote regions, the accounts of this genus were constantly added (e.g., Cai et al., 2020; Du et al., 2021, 2022; Yang et al., 2017, 2019). During a field investigation on a sclerophyllous oak community (dominated by *Quercus guyavifolia* H.Lév.) in August 2021, we found an unknown species of *Oreocharis* in Ninglang County, North Yunnan Province. It matches the morphological commonalities of *Oreocharis*, with characters such as rosette habit, bilateral symmetrical flowers, four fertile stamens, and capsule loculicidal dehiscing into two valves right down the receptacle (see Table S1). It resembles both *O. georgei* Anthony (1934: 202) and *O. forrestii* (Diels) Skan (1917: t. 8719), but it fits neither of them with its unique characters, such as venation on leaves, indumentum of peduccle, pedicel and bract, and the shape of bract. It was confirmed to be a new species by our further literature perusal (Pan et al., 2019; Wang et al., 1998; Yang et al., 2017) and morphological comparison with herbarium specimens (physical and virtual specimens at BM, CDBI, E, FI, IBK, IBSC, K, KUN, P, PE, and US, acronym following Thiers, 2022). And it is thereafter named as *O. oriolus*. Phylogenetic analysis using mainly nuclear ribosome internal transcribed spacer (nrITS) and chloroplast DNA fragment (*trnL‐F*) confirmed its position in this genus.

## 2 MATERIALS AND METHODS

### 2.1 Morphological description

The measurements and description of the new species, *Oreocharis oriolus*, were based on living plants in the field and the specimens at herbarium (CDBI & IBK), the morphological characters were measured using ImageJ v1.53k (Schneider et al., 2012) and are described using the terminology proposed by Harris and Harris (1994), Wang et al. (1998). Studied voucher specimens including the type materials and additional silica‐gel dried leaves are stored at CDBI and IBK.

### 2.2 DNA extraction, amplification, and sequencing

The sequences of the 67 species for molecular phylogenetic analysis were retrieved from GenBank (accessions referring to Table S2). A total of 62 species of *Oreocharis* was used as ingroup and five species from two genera (four specie of *Agalmyla* Blume (1826: 766) and one species of *Metapetrocosmea* Wang (1981: 38)) as outgroup **(**Lv et al., 2022). The *nr*ITS and *trnL‐F* sequences of *Oreocharis forrestii* (a morphologically closely related species of *O. oriolus*) were extracted from the transcriptome (GenBank accession: SRR12339718) of this species published by Kong et al. (2022) using Easy353 v1.5.0 (Zhang et al., 2022) with default parameters, owing to its unavailability of leaf materials. The sequences of *O. oriolus* were newly generated as follows. Total DNA was extracted exclusively from silica‐gel dried leaves using a Plant DNA Isolation Kit (Cat. No. DE‐06111, Foregene, Chengdu, China). The primers used by Yang et al. (2020) and Lv et al. (2022) were adopted. The nuclear ribosomal internal transcribed spacer (*nr*ITS) and chloroplast DNA fragment (*trnL‐F*) were amplified by polymerase chain reaction (PCR). All DNA samples were sent to TSINGKE Biotech Co. Ltd (Chengdu, China) for sequencing and then deposited to GenBank under the accession numbers: ON869242 and ON809546, respectively (Table S2).

### 2.3 Phylogenetic analyses

The sequences of the new species were processed using Sequencher v4.1.4 (Gene Codes, Ann Arbor, Michigan, USA) and aligned with all used sequences via MAFFT v7.475 (Katoh & Standley, 2013) with default parameters. The incongruence length difference test (ILD) was used to quantify the conflicts between nuclear DNA and plastid DNA data in PUPA v4.0a169 (Darlu & Lecointre, 2002; Swofford, 2002). Maximum likelihood (ML) and Bayesian inference (BI) methods were applied to infer the gene tree. jModeltest v2.1.6 (Posada, 2008) identified GTR+G as the best model for *nr*ITS and *trnL‐F* which selected using the corrected Akaike Information Criterion (AICc). BI analysis was conducted using MrBayes 3.2.7a (Ronquist & Huelsenbeck, 2003) with two parallel runs (10 million generations). The first 25% of the trees were discarded as burn‐in, and the remaining trees were used to generate a majority‐rule consensus tree. ML analysis was performed using IQ‐TREE v1.4.241 (Nguyen et al., 2014), the ModelFinder in IQ‐TREE tested a total of 286 DNA models and chose GTR+F+I+G4 as the best‐fit nucleotide substitution model for the combined *nr*ITS and *trnL‐F* matrices, and branch support was estimated using 2000 replicates of ultrafast bootstrapping algorithm (UFboot) (Minh et al., 2013).

## 3 RESULTS

**FIGURE 1:**
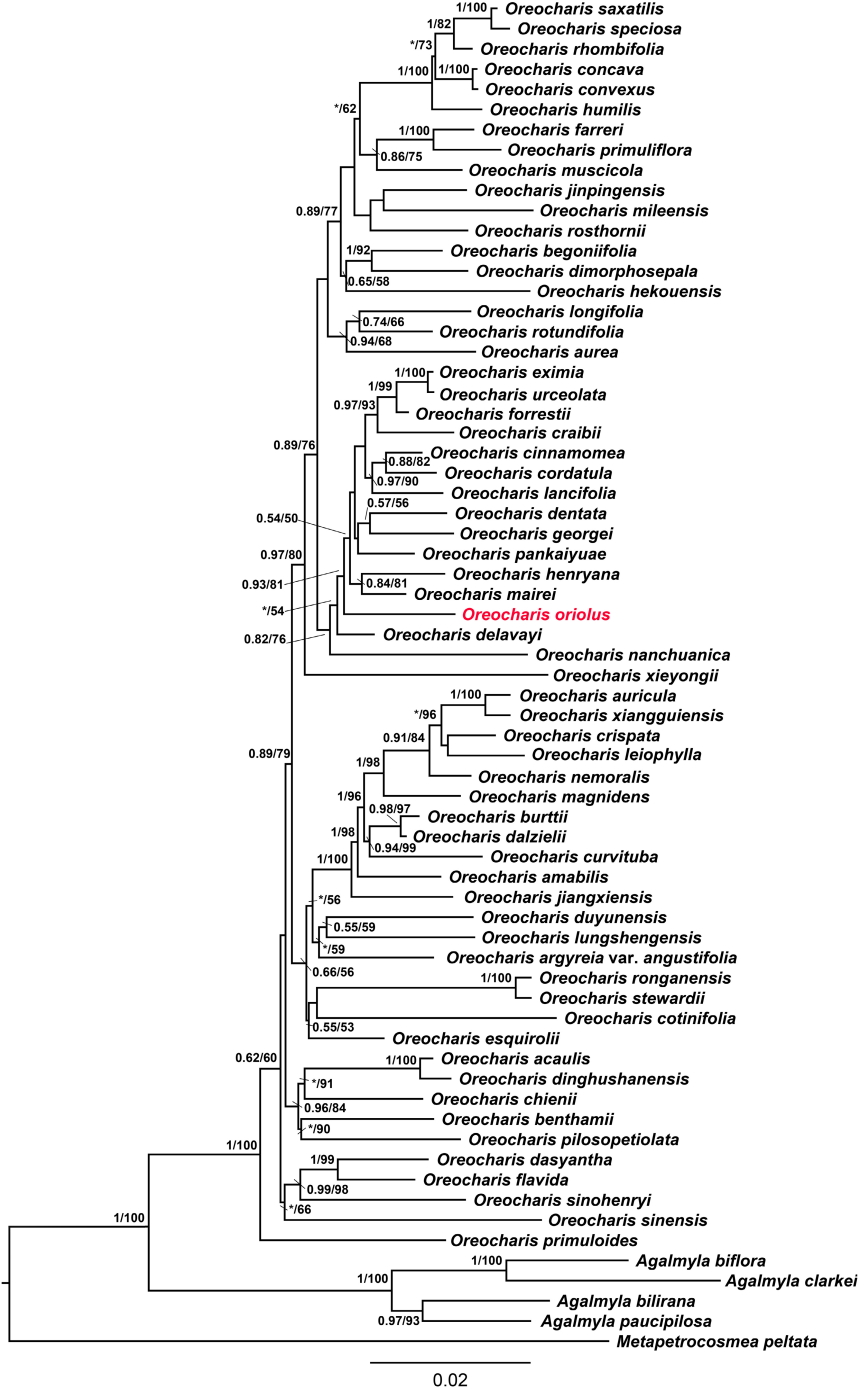
Bayesian phylogenetic tree inferred from combined *nr*ITS and *trnL‐F* sequences data. Values above branches are maximum likelihood bootstrap percentages (>50)\Bayesian posterior probabilities (>.5), “*” denotes branches with <50% bootstrap support. The new species is shown in red. Species names of *Oreocharis* follow Möller et al. (2011a, 2011b).

The ILD test (*p* = .6) showed that the plastid DNA (*trnL‐F*) and nuclear DNA (*nr*ITS) were highly congruent. The molecular phylogenetic tree showed that the 62 species of *Oreocharis* formed a well‐supported monophyletic group (BI/ML = 1/100, Figure 1). The inferred new species clustered with other 12 species, including both *O. georgei* and *O. forrestii*, into a subclade with relatively well support (BI/ML = 0.93/81, Figure 1), and which further consisted a clade by incorporating *O. delavayi* Franchet (1888: 715) and *O. nanchuanica* (Pan & Liu, 1995: 100) Mich.Möller & A. Weber in Möller et al. (2011a, 2011b: 23) (BI/ML = 0.82/76, Figure 1).

## 4 TAXONOMIC TREATMENT

**FIGURE 2:**
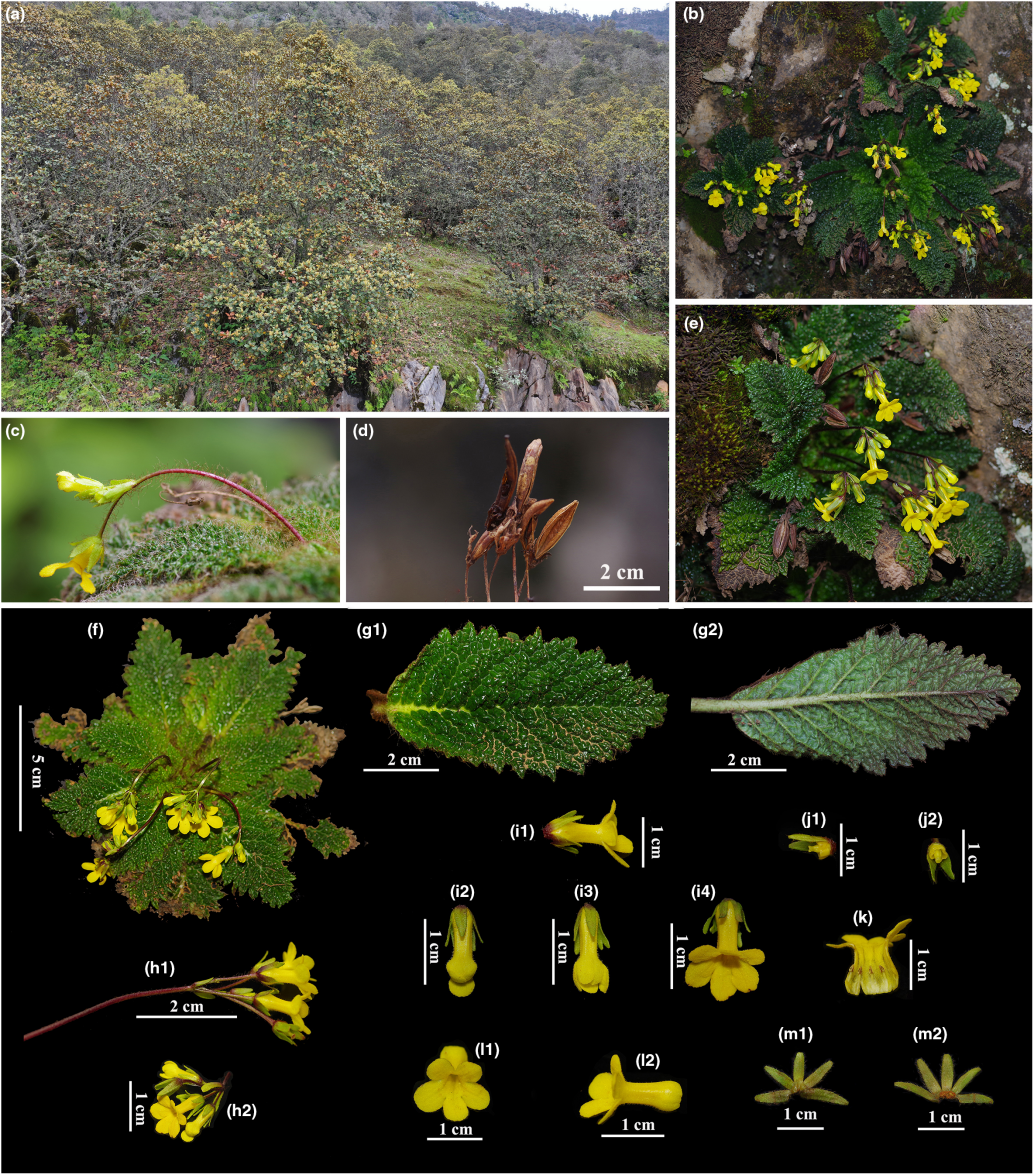
*Oreocharis oriolus*. (a) Habitat; (b) A subpopulation; (c) Cyme and indumentum of peduncle; (d) Infructescence of previous year; (e and f) Habit; (g‐1) Adaxial leaf surface; (g‐2) Abaxial leaf surface; (h‐1) Side view of cyme; (h‐2) Front view of cyme; (i‐1) Side view of flower; (i‐2, i‐3, i‐4) Flowers at different opening stages; (j‐1) Side view of ovary; (j‐2) Front view of ovary; (k) Opened corolla, showing stamens and staminodes; (l‐1) Front view of corolla; (l‐2) Side view of corolla; (m‐1) Abaxial surface of calyx; (m‐2) Inner surface of calyx.

**FIGURE 3:**
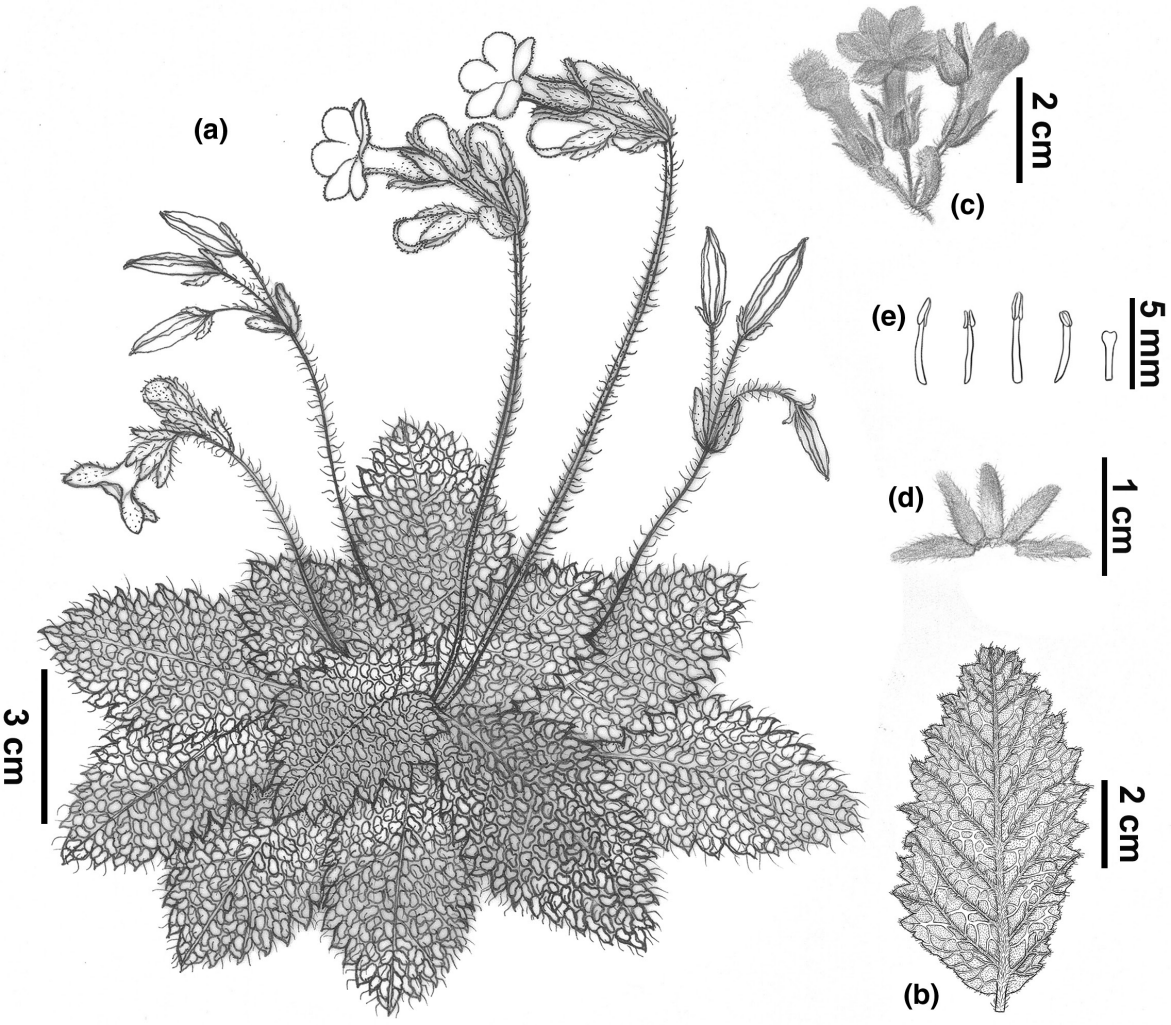
*Oreocharis oriolus*. (a) Habit; (b) Abaxial leaf; (c) Cyme; (d) Calyx; (e) Fertile stamens (left four) and a staminode (right). Drawn by Yu‐Jie Chen.

***Oreocharis oriolus*** J. Hu & F. Wen, sp. *nov*. (Figures 2 and 3).

### 4.1 Type

CHINA. Yunnan: Ninglang County, Guanguanshan Village, Yizu Ditch, growing in limestone cracks, 27.2664°N, 100.6208°E, elev. ca. 3410 m, 23 July 2021, *J. Hu* et al. *hujun20210723B02* (holotype CDBI!; isotypes CDBI!, IBK!).

### 4.2 Diagnosis

It is morphologically similar to *Oreocharis forrestii*, but it can be distinguished from the latter in bracts lanceolate (vs. linear to linear‐lanceolate) and adaxial surface glabrescent to nearly glabrous (vs. sparsely rust‐brown villous or pubescent), pedicel sparsely brownish pubescent (vs. villous and glandular‐pubescent), calyx lobes glabrescent to nearly glabrous on abaxial surfaces (vs. sparsely pubescent and glandular‐puberulent), anthers ovate‐triangle to nearly triangle (vs. broadly oblong).

### 4.3 Description

Perennial acaulescent herb with rhizomatous short stem 2–4 mm long and 8–12 mm in diameter. **Leaves** in basal rosette, 6–12 or rarely more; petioles 5–20(−40) mm long, 3.5–4.5 mm in diameter, pale green to green, densely rust‐brown villous; **leaf blade** ovate to elliptic, 4–10 × 2.5–5 cm, base cuneate to broadly cuneate, symmetric or slightly asymmetric, apex obtuse to acute, margin irregularly serrate, rarely biserrate and with rusty ciliate, adaxial surface green with veinlets depressed and forming bullate networks, abaxial surface pale green, rusty villous, mixed rusty and whitish pubescent with veinlets prominent; lateral veins 7–11 pairs, obviously depressed adaxially and elevated abaxially. **Cymes** axillary, 2–4, (1–)2–6(−10)‐flowered; **peduncle** brownish red, 5–10 (−12) cm long, 1–1.3 mm in diameter, densely mixed rusty villous, hairs slightly curly, 3–4 mm long; **bracts** 2, oblanceolate to oblong‐oblanceolate, 4–8 × ca. 1 mm, apex acute to obtuse, margin entire, adaxially green and glabrescent, abaxially yellowish green but from apex to base gradually transitioning to pale brownish red and glabrous, margin sparsely ciliate; **pedicel** pale brownish red, 1–3 cm long, ca. 0.8 mm in diameter, sparsely brownish pubescent; **calyx** 5‐lobed to base, lobes nearly equal in shape and size, oblong‐lanceolate to broadly lanceolate, 4–6 × ca. 1.5 mm, green but from apex to base gradually transitioning to pale brownish, apex acute to acuminate, margin entire, or occasionally one or more inconspicuously serrate near the apex on each side, both surfaces glabrescent; **corolla** brightly yellow, ca. 1.5 cm long, outside sparsely glandular‐puberulent, inside glabrous, corolla tube ca. 10 mm long, narrowly cylindric while gradually enlarging from throat to base and forming slightly swollen pot‐bellied shape, ca. 4 mm in diameter at the middle of the tube, throat slightly constricted; limb distinctly 2‐lipped, adaxial lip 2‐lobed, lobes ovate, 3–4 × ca. 3.5 mm, abaxial lip 3‐lobed, longer than adaxial lip, lobes ovate, 4–5 × ca. 4 mm; **stamens** free, included, all adnate to ca. 2 mm above the base of the corolla tube, free filaments to 2 mm long, pale yellow to white, glabrous, **anthers** ovate‐triangle to nearly triangle, ca. 1.2 mm long, locules 2, confluent at apex; **staminode** present, ca. 3.5 mm long; **disc** yellow, ca. 1 mm in height, margin undulant, glabrous; **pistil** included, ca. 4.2 mm long, glabrous, ovary oblong, ca. 3 mm long, 1.2 mm in diameter, style ca. 1.2 mm long, 0.9 mm in diameter, stigma 1, glabrous, discoid, centrally depressed. **Capsule** brown, oblong‐elliptic to narrowly elliptic, 1.5–3.5 cm long, 4–5 mm in diameter, glabrous, loculicidal dehiscing predominantly on one side.

### 4.4 Phenology

Flowering in July and fruiting from August to November.

### 4.5 Etymology

The brightly yellow flowers of this new species reminisced about the lively birds oriole (*Oriolus oriolus*), and the epithet is here used as a noun in apposition. Its Chinese name, Huáng Lí Mǎ Líng Jù Taí (黄鹂马铃苣苔), is also taken this envision.

### 4.6 Distribution and habitat

**FIGURE 4:**
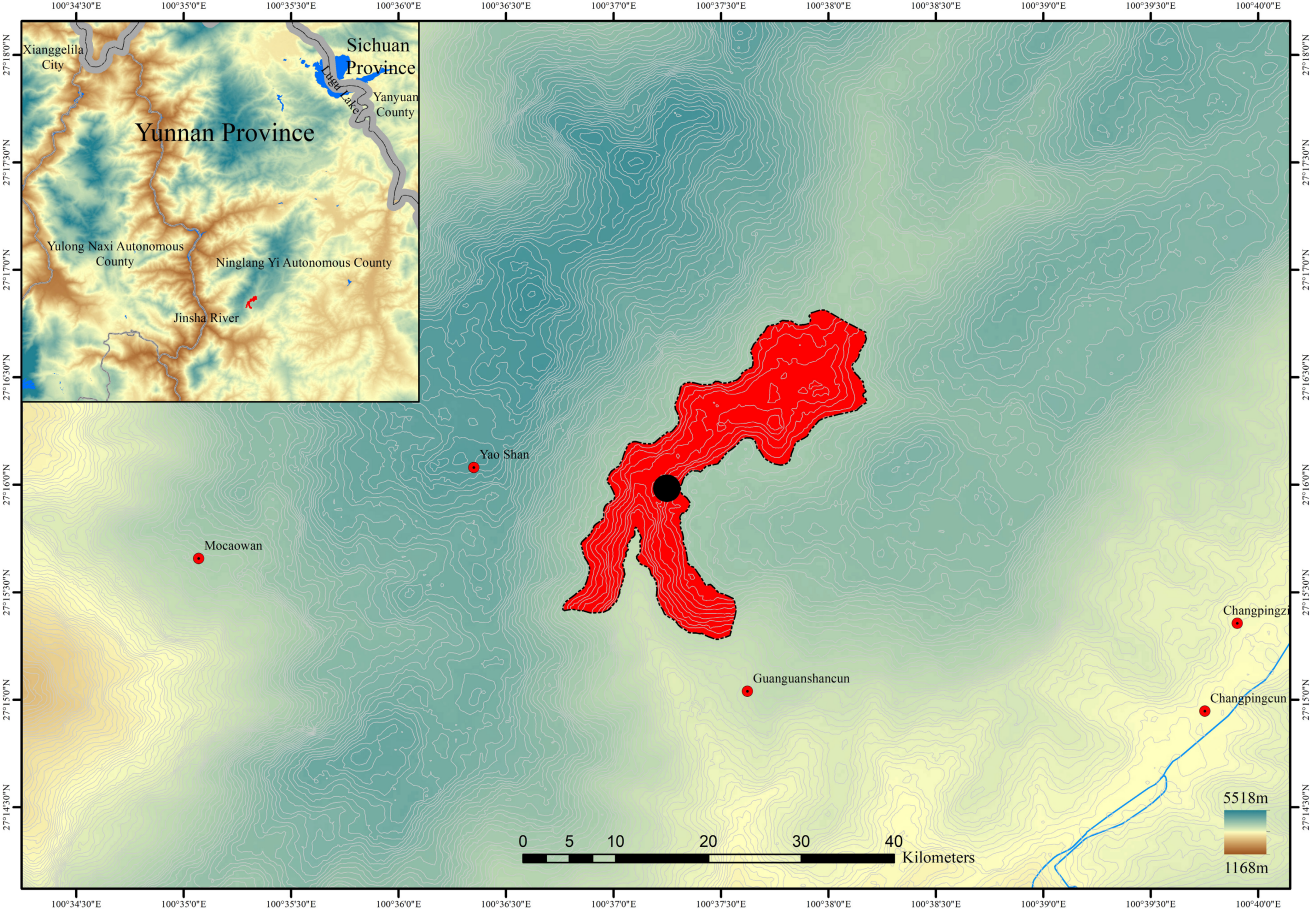
Distribution map of *Oreocharis oriolus* (Red area: scope of distribution; Black dot: location of specimen collected).

This new species is presently only known from type locality, North Yunnan, Southwest China (detailed referring to the type specimen citation, Figure 4). It grows under one of our investigated subalpine sclerophyllous oak communities (dominated by *Quercus guyavifolia* H. Lév.), mostly restricted in a limestone crevice, where it is accompanied by *Aleuritopteris kuhnii* (Milde) Ching, *Allium mairei* H. Lév., *Meeboldia delavayi* (Franch.) W. Gou & X. J. He, *Paragymnopteris vestita* (Wall. ex C.Presl) K. H. Shing, *Saxifraga rufescens* Balf. f., *Sedum chauveaudii* Raym.‐Hamet, *Tongoloa rockii* H. Wolff, and an unidentified species of *Woodsia* R. Br.

### 4.7 Conservation status

Apart from the subpopulation where we did the vegetation survey and the type specimen was collected, there are approximately 300 individuals confirmed restricted in an area of 2 km^2^ (Figure 4). We also made extensive vegetation investigations in adjacent areas and failed to find this new species at similar habitats. It is temporarily assessed as Critically Endangered (CR B2ab (iii, v)) based on the IUCN red list of Threatened Species (IUCN, 2021) to highlight its conservation emergency.

### 4.8 Taxonomic affinities

The corolla of being campanulate tubular and the length of tube being about two times that of corolla limb showed that *Oreocharis oriolus* should be considered as a morphologically most related species of *O. forrestii*. And this new species also resembles *O. georgei* in dichotomous cyme with 4–10 flowers, in narrowly cylindrical corolla tube slightly constricted at throat while moderately inflated at base, in 2‐lipped corolla limb equal to or slightly shorter than corolla tube in length, and in usually 2‐sect adaxial lip and 3‐sect abaxial lip with lobes oblong to oblong‐lanceolate. A comparison of morphological characters of these three species are summarized in Table 1.

**TABLE 1:** Morphological comparison among *Oreocharis oriolus*, *O. forrestii* and *O. georgei*.

Characters	*O. oriolus*	*O. forrestii*	*O. georgei*
Leaf blade			
Adaxial surface	Bullate; rust‐brown and white pubescent	Slightly and indistinctive bullate; sparsely rust‐brown villous and white pubescent	Smooth, whitish pubescent, with a few rust‐brown hairs
Abaxial surface	Main, lateral and secondary veins obviously prominent and forming hollows; rust‐brown and white pubescent	Densely rust‐brown villous and white pubescent	Densely rust‐brown villous, glabrescent between veins
Peduncle indumentum	Rusty villous, mixed with rusty and whitish short hairs	Sparsely to densely rust‐brown villous and pubescent	Rust‐brown villous
Bract			
Shape	Lanceolate	Linear to linear‐lanceolate	Linear
Indumentum	Adaxially glabrescent to nearly glabrous	Adaxially sparsely pubescent and glandular‐puberulent	Adaxially rust‐brown villous
Corolla limp			
Adaxial lip lobes shape	Ovate	Semicircular	Nearly rounded
Adaxial lip lobes size	3–4 × ca. 3.5 mm	1–2 × 1–2 mm	2–3 × 1.5–2 mm
Stamens			
Places in corolla tube	All places at equal height, adnate to ca. 2 mm above the base of the corolla tube	Two pairs placed at different height, epigynous pair adnate to ca. 2 mm and hypogynous pair adnate to ca. 1.5 mm above the base of the corolla tube	Two pairs placed at different height, epigynous pair adnate to ca. 2 mm and hypogynous pair adnate to ca. 3 mm above the base of the corolla tube
Free filaments length	All to 2 mm long	Epigynous stamens' filaments ca. 3 mm long, hypogynous stamens' filaments ca. 4 mm long	All ca. 4 mm long
Ovary	Oblong, ca. 3 mm long	Ovate, ca. 4 mm long	Ovate, ca. 4 mm long

## AUTHOR CONTRIBUTIONS

**Jun Hu:** Formal analysis (lead); investigation (lead). **Junyi Zhang:** Formal analysis (supporting); methodology (lead). **Hai He:** Formal analysis (supporting); writing – review and editing (supporting). **Ding‐xiang Yu:** Visualization (supporting); writing – review and editing (equal). **Hong Jiang:** Investigation (supporting). **Qing Liu:** Funding acquisition (supporting); project administration (lead); writing – review and editing (equal). **Fang Wen:** Formal analysis (supporting); writing – original draft (supporting); writing – review and editing (supporting).

## FUNDING INFORMATION

This study is jointly supported by the Second Tibetan Plateau Scientific Expedition and Research (STEP) program (Grant No. 2019QZKK0301), Major Program for Basic Research Project of Yunnan Province (202101BC070002), the Key Sci. & Tech. Research and Development Project of Guangxi (Guike AD20159091 & ZY21195050), the capacity‐building project of SBR of CAS (KFJ‐BRP‐017‐68), the Foundation of Guangxi Key Laboratory of Plant Conservation and Restoration Ecology in Karst Terrain (22‐035‐26) and the Basic Research Fund of Guangxi Academy of Sciences (grant no.CQZ‐C‐1901).

## CONFLICT OF INTEREST STATEMENT

There is no conflict of interest to declare.

## Supporting information


Table S1

Table S2

## Data Availability

DNA sequences: GenBank accessions: ON869242 and ON809546. Appendix Table to this article can be found online.
