# Supplementary material for: Oreocharis oriolus, a new species of Gesneriaceae in a sclerophyllous oak community from Yunnan, Southwest China
Source: Ecol Evol. 2023 Jun 26;13(6):e10174. doi: 10.1002/ece3.10174 (PMC10293703; doi:10.1002/ece3.10174)
Supplement: Supplementary file 1 — Table S1 [file ECE3-13-e10174-s002.docx]

**Table S1.**  Character matrix of *Oreocharis*.

| Species name | Corolla color | Lip length | Stamen | Species name | Corolla color | Lip length | Stamen |
| --- | --- | --- | --- | --- | --- | --- | --- |
| *Oreocharis auricula* | 0 | 1 | 0 | *Oreocharis pankaiyuae* | 2 | 2 | 1 |
| *Oreocharis xiangguiensis* | 0 | 1 | 0 | *Oreocharis primuliflora* | 0 | 1 | 0 |
| *Oreocharis crispata* | 0 | 2 | 0 | *Oreocharis farreri* | 0 | 1 | 0 |
| *Oreocharis leiophylla* (*Bournea leiophylla*) | 1 | 1 | 1 | *Oreocharis dentata* | 2 | 2 | 0 |
| *Oreocharis nemoralis* | 0 | 1 | 0 | *Oreocharis henryana* | 0 | 1 | 0 |
| *Oreocharis lungshengensis* | 0 | 2 | 0 | *Oreocharis jinpingensis* | 3 | 2 | 0 |
| *Oreocharis magnidens* | 0/3 | 1 | 0 | *Oreocharis longifolia* | 1 | 2 | 0 |
| *Oreocharis burttii* | 0 | 2 | 0 | *Oreocharis rotundifolia* | 1 | 2 | 0 |
| *Oreocharis dalzielii* | 0 | 2 | 0 | *Oreocharis benthamii* | 0 | 1 | 0 |
| *Oreocharis curvituba* | 0 | 2 | 0 | *Oreocharis aurea* | 1 | 2 | 0 |
| *Oreocharis esquirolii* | 0 | 1 | 1 | *Oreocharis nanchuanica* | 1 | 1 | 0 |
| *Oreocharis jiangxiensis* | 0 | 2 | 1 | *Oreocharis dinghushanensis* | 0 | 2 | 0 |
| *Oreocharis argyreia* | 0 | 1 | 0 | *Oreocharis acaulis* | 0 | 2 | 0 |
| *Oreocharis flavida* | 1 | 1 | 0 | *Oreocharis dasyantha* | 2 | 2 | 0 |
| *Oreocharis concava* | 2 | 2 | 0 | *Oreocharis hekouensis* | 1 | 2 | 0 |
| *Oreocharis convexa* | 2 | 2 | 0 | *Oreocharis mileensis* | 1 | 1 | 0 |
| *Oreocharis saxatilis* | 1 | 2 | 0 | *Oreocharis primuloides* | 0 | 2 | 0 |
| *Oreocharis speciosa* | 0 | 2 | 0 | *Oreocharis pilosopetiolata* | 0/3 | 2 | 1 |
| *Oreocharis humilis* | 1 | 2 | 0 | *Oreocharis amabilis* | 0 | 1 | 0 |
| *Oreocharis rhombifolia* | 1 | 1 | 0 | *Oreocharis chienii* | 0 | 2 | 0 |
| *Oreocharis rosthornii* | 0 | 2 | 0 | *Oreocharis cotinifolia* | 0/3 | 1 | 1 |
| *Oreocharis mairei* | 3 | 2 | 1 | *Oreocharis sinohenryi* | 0 | 2 | 0 |
| *Oreocharis delavayi* | 1 | 2 | 1 | *Oreocharis xieyongii* | 0 | 0 | 1 |
| *Oreocharis eximia* | 1 | 1 | 0 | *Oreocharis duyunensis* | 0 | 2 | 0 |
| *Oreocharis urceolata* | 1 | 2 | 1 | *Oreocharis stewardii* | 0 | 2 | 0 |
| *Oreocharis cinnamomea* | 1 | 1 | 0 | *Oreocharis ronganensis* | 0 | 2 | 0 |
| *Oreocharis cordatula* | 1 | 1 | 0 | *Oreocharis sinensis* (*Bournea sinensis*) | 3 | 1 | 1 |
| *Oreocharis craibii* | 1 | 2 | 1 | ***Oreocharis orilus*** | 0 | 2 | 1 |
| *Oreocharis begoniifolia* | 3 | 2 | 1 | *Metapetrocosmea peltata* | 3 | 2 | 0 |
| *Oreocharis dimorphosepala* | 0 | 2 | 0 | *Agalmyla biflora* | 4 | 1 | 1 |
| *Oreocharis forrestii* | 0 | 2 | 1 | *Agalmyla bilirana* | 4 | 1 | 0 |
| *Oreocharis georgei* | 1 | 2 | 0 | *Agalmyla paucipilosa* | 4 | 1 | 0 |
| *Oreocharis muscicola* | 2 | 2 | 0 | *Agalmyla clarkei* | 5 | 1 | 1 |
| *Oreocharis lancifolia* | 0 | 1 | 0 |  |  |  |  |
