# Supplementary material for: Oreocharis oriolus, a new species of Gesneriaceae in a sclerophyllous oak community from Yunnan, Southwest China
Source: Ecol Evol. 2023 Jun 26;13(6):e10174. doi: 10.1002/ece3.10174 (PMC10293703; doi:10.1002/ece3.10174)
Supplement: Supplementary file 2 — Table S2 [file ECE3-13-e10174-s001.docx]

**Table S2.** The GenBank accessions for DNA sequences (ITS and *trn*L-F) used in this paper.

| Species Name | ITS | *trn*L-F | Species Name | ITS | *trn*L-F |
| --- | --- | --- | --- | --- | --- |
| *Oreocharis auricula* | DQ912664 | FJ501481 | *Oreocharis pankaiyuae* | HQ633021 | HQ632925 |
| *Oreocharis xiangguiensis* | HQ633027 | HQ632932 | *Oreocharis primuliflora* | HQ633019 | JF697585 |
| *Oreocharis crispata* | MN164507 | MN164493 | *Oreocharis farreri* | JF697573 | JF697585 |
| *Oreocharis leiophylla* | GU350644 | GU350676 | *Oreocharis dentata* | KM063156 | KM062916 |
| *Oreocharis nemoralis* | MN164498 | MN164490 | *Oreocharis henryana* | JF697574 | KM062918 |
| *Oreocharis lungshengensis* | HQ633031 | HQ632917 | *Oreocharis jinpingensis* | KM063163 | KM062923 |
| *Oreocharis magnidens* | HQ633026 | HQ632930 | *Oreocharis longifolia* | KM063152 | KM062912 |
| *Oreocharis burttii* | JF697570 | JF697582 | *Oreocharis rotundifolia* | KM063151 | KM062911 |
| *Oreocharis dalzielii* | JF697571 | JF697583 | *Oreocharis benthamii* | JF697572 | JF697584 |
| *Oreocharis curvituba* | MN164500 | MN164491 | *Oreocharis aurea* | HQ633016 | KM062914 |
| *Oreocharis esquirolii* | HQ633011 | HQ632915 | *Oreocharis nanchuanica* | KM063164 | KM062924 |
| *Oreocharis jiangxiensis* | HQ633029 | HQ632933 | *Oreocharis dinghushanensis* | GU350643 | GU350675 |
| *Oreocharis argyreia* | GU350639 | GU350671 | *Oreocharis acaulis* | HQ633012 | HQ632916 |
| *Oreocharis flavida* | MF315099 | MF315105 | *Oreocharis dasyantha* | MK587955 | MF315104 |
| *Oreocharis concava* | FJ501336 | FJ501505 | *Oreocharis hekouensis* | KM063174 | KM062934 |
| *Oreocharis convexa* | FJ501337 | FJ501506 | *Oreocharis mileensis* | KM062943 | MK342624 |
| *Oreocharis saxatilis* | KM063171 | KM062932 | *Oreocharis primuloides* | FJ501364 | FJ501546 |
| *Oreocharis speciosa* | KJ475420 | KM232646 | *Oreocharis pilosopetiolata* | MH629755 | MH629762 |
| *Oreocharis humilis* | GU350633 | GU350665 | *Oreocharis amabilis* | KJ47433 | KM232654 |
| *Oreocharis rhombifolia* | GU350632 | GU350664 | *Oreocharis chienii* | KM063148 | KM062908 |
| *Oreocharis rosthornii* | KM063167 | KM062927 | *Oreocharis cotinifolia* | HQ633010 | MN579510 |
| *Oreocharis mairei* | GU350658 | GU350689 | *Oreocharis sinohenryi* | HQ633009 | HQ632913 |
| *Oreocharis delavayi* | KM063155 | KM062915 | *Oreocharis xieyongii* | MN783374 | MN786528 |
| *Oreocharis eximia* | KM063159 | KM062919 | *Oreocharis duyunensis* | MG722856 | MG722858 |
| *Oreocharis urceolata* | HQ633018 | HQ632922 | *Oreocharis stewardii* | HQ633022 | HQ632926 |
| *Oreocharis cinnamomea* | KM063161 | KM062921 | *Oreocharis ronganensis* | HQ633023 | HQ632927 |
| *Oreocharis cordatula* | KM063162 | KM062922 | *Oreocharis sinensis* | HQ633008 | HQ632912 |
| *Oreocharis craibii* | HQ633017 | HQ632921 | ***Oreocharis oriolus*** | **ON869242** | **ON809546** |
| *Oreocharis begoniifolia* | HQ633025 | KM062926 | *Agalmyla biflora* | FJ501361 | FJ501541 |
| *Oreocharis dimorphosepala* | KM063165 | KM062925 | *Agalmyla bilirana* | MF446046 | HQ632891 |
| *Oreocharis georgei* | KM063157 | KM062917 | *Agalmyla paucipilosa* | HQ632990 | HQ632893 |
| *Oreocharis muscicola* | DQ912665 | FJ501548 | *Agalmyla clarkei* | FJ501360 | FJ501540 |
| *Oreocharis lancifolia* | HQ633020 | HQ632924 | *Metapetrocosmea peltata* | HQ632968 | HQ632872 |
